# Transarterial Chemoembolization with BioPearls for the Treatment of Hepatocellular Carcinoma: A Preliminary Experience

**DOI:** 10.3390/ph18030307

**Published:** 2025-02-23

**Authors:** Roberto Iezzi, Alessandro Posa, Irene Bargellini, Carlo Spreafico

**Affiliations:** 1 Emergency and Interventional Radiology Unit, Department of Diagnostic Imaging and Oncologic Radiotherapy, Fondazione Policlinico Universitario “Agostino Gemelli”-IRCCS, 00168 Rome, Italy; 2 Facoltà di Medicina e Chirurgia, Università Cattolica del Sacro Cuore, 00168 Rome, Italy; 3 Division of Diagnostics and Interventional Radiology, Candiolo Cancer Institute FPO-IRCCS, 10060 Candiolo, Italy; irenebargellini@hotmail.com; 4 Department of Surgical Sciences, University of Turin, 10126 Turin, Italy; 5 Department of Radiology, Fondazione IRCCS Istituto Nazionale Tumori di Milano, 20133 Milan, Italy; carlo.spreafico@istitutotumori.mi.it

**Keywords:** hepatocellular carcinoma, transarterial chemoembolization, safety, tumor response, doxorubicin, drug-eluting microspheres

## Abstract

**Background/Objectives**: Transarterial chemoembolization (TACE) is a widely accepted and minimally invasive treatment for primary and metastatic liver cancer. Performing TACE with drug-eluting beads helps obtain a greater drug concentration in the target lesion, significantly reducing systemic drug leakage, liver toxicity, and adverse events. The aim of this study is to describe the safety and feasibility of TACE performed with BioPearl^TM^, the first biodegradable drug-eluting microspheres. **Methods**: This was a retrospective observational study on 13 consecutive patients affected by hepatocellular carcinoma (HCC) treated with doxorubicin-loaded-BioPearl^TM^-TACE. Data on safety, feasibility, and tumor response were collected. **Results**: One intra-procedural catheter blockage was registered, as well as two post-treatment bilomas that required additional treatment. No severe general drug-related side effects were detected at the follow-up. The 1-month overall disease control was 90.9%, with six complete responses. **Conclusions**: Data suggest that chemoembolization with BioPearl^TM^ is feasible and safe for the treatment of HCC as indicated by good tolerability.

## 1. Introduction

The hepatocellular carcinoma (HCC) is one of the most common neoplasms, ranking sixth and third among all tumors in the world in terms of incidence and mortality, respectively, with an incidence rate that is increasing in a great number of countries across Europe, North America, Australia and New Zealand, and South America [1]. Unfortunately, HCC patients are often diagnosed in intermediate and advanced stages, in which curative options are limited due to both lesion number and liver impairment [2]. Among locoregional therapies, when curative treatment options (i.e., surgical resection, percutaneous ablation, and transarterial radioembolization) are not indicated, transarterial chemoembolization (TACE) can be considered [2]. TACE is a widely accepted and minimally invasive treatment for patients with HCC, as studies have confirmed that TACE can prolong the overall survival of patients with intermediate-stage HCC [2,3,4,5,6,7]. Moreover, TACE can also be used in patients with an early-stage HCC in case of refusal of surgery or an inability to perform more curative treatments, such as a down-staging technique, or as a bridge to curative treatments [2]. Classically, TACE was performed using an emulsion of iodinated oil and a chemotherapeutic drug, the so-called conventional TACE (cTACE), but it was hindered by drug-related systemic adverse events due to drug leakage from the target HCC lesion to the systemic circulation. The advent of drug-eluting beads TACE (DEB-TACE) significantly increased the efficiency of drug delivery to the target neoplastic lesion during this procedure, while reducing systemic diffusion, liver toxicity, and adverse events [3,4,5,6,7]. There have been many studies in the literature reporting on both the clinical advantages and shortcomings of DEB-TACE compared to cTACE [3,4,5,6,7]. The most common adverse event related to DEB-TACE is represented by postembolization syndrome, which is related to the embolic and ischemic effect of the beads released in tumor vasculature and includes fatigue, pain, fever, nausea and vomiting, and increase in liver transaminases; moreover, the occlusion of the arterial vessels by the DEBs can lead to bile duct injury, liver bilomas, and abscess formation, up to liver function impairment and failure [7,8]. The use of permanent beads can, therefore, severely affect the functioning of an already impaired cirrhotic liver, leading to severe clinical sequelae [7,8]. Moreover, the permanent occlusion of the arterial vessel feeding the HCC lesion, obtained with the use of DEB-TACE, determines tissue ischemia and the consequent release of cytokines and growth factors in the bloodstream that lead to neo-angiogenesis and the formation of parasite vascularization to the neoplastic lesion; these aberrant and newly formed vessels are thin and prone to spasm during TACE, and they can originate from non-conventional arterial branches (i.e., diaphragmatic, cystic, and renal and adrenal arteries), thereby increasing the difficulty of subsequent future TACE retreatments [9,10]. Another TACE technique is the one based on degradable starch microspheres (DSM-TACE); these microparticles are degraded in the bloodstream by alpha-amylases, causing only a temporary occlusion of the feeding vessel while delivering the chemotherapeutic agent deeply in the target lesion and granting rapid vessel reperfusion [4]. However, as the starch microspheres do not bind to the chemotherapeutic agent but are only mixed with it in a contrast medium-based solution, there is no actual loading of the chemotherapeutic agent; this leads to a rapid and non-controlled release of the drug into the liver bloodstream [4].

The aim of this study is to retrospectively report the safety and feasibility of DEB-TACE performed with BioPearl^TM^ microspheres, the first biodegradable drug-eluting microspheres. These microspheres can be loaded with the chemotherapeutic agent and can release it in a controlled and sustained manner. On the other hand, being resorbable, these microspheres can lead to a progressive restoration of the blood flow in the arterial vessel feeding the neoplastic lesion for future intra-arterial treatments, thereby overcoming the limitations of permanent microparticles.

## 2. Results

### 2.1. Sample Description

The sample population included 13 consecutive patients affected by HCC with a multidisciplinary tumor board indication to undergo TACE treatment. Among these, 11 patients (84.6%) were males, while 2 patients (15.4%) were females. The average patient age was 74.6 ± 9.8 years (range 56–83). The average number of lesions treated was 1.7 ± 1.2 (range 1–5), with a total number of 21 treated lesions. The maximum diameter of the lesions was smaller than 5 cm in size in 84.6% of patients (11/13). Meanwhile, 11 out of 13 patients (84.6%) had a unilobar disease; of these, 5 patients (45.5%) had the disease in the right liver lobe, whereas 6 patients (54.5%) had disease in the left liver lobe. Only two patients (15.4%) had a bilobar disease. HCC lesions were mainly (66.6%) located in segments IV, VI, and VII. All patients had a Child–Pugh class A liver disease. All patients were treatment-naïve and had an ECOG-PS of 0. Ten patients (76.9%) had an early (BCLC stage A) HCC, while three patients (23.1%) had an intermediate (BCLC stage B) HCC. Indication for TACE was curative in 69.2% of cases (9/13 patients), while in the other 4 patients (30.8%), the TACE procedure was indicated for down-staging (7.7%), bridging (7.7%), and palliation (15.4%) purposes (Table 1).

### 2.2. Feasibility and Efficacy

Technical success, defined as the complete administration of the BioPearl^TM^ microspheres loaded with doxorubicin in the target HCC lesion, was achieved in 100% of patients. The median follow-up was 24 months; 11/13 patients (84.6%) were evaluated 1 month after TACE, with CT or MRI using the modified Response Evaluation Criteria in Solid Tumors (mRECIST) [11]. Six patients (54.5%) had a complete response (CR), four (36.4%) had a partial response (PR), and only one (9.1%) patient showed disease progression (PD); the overall disease control (ODC) rate was 90.9%. One patient died 3 weeks after the treatment for reasons not related to the TACE treatment, whereas the last patient was lost in the follow-up.

**Figure 1:**
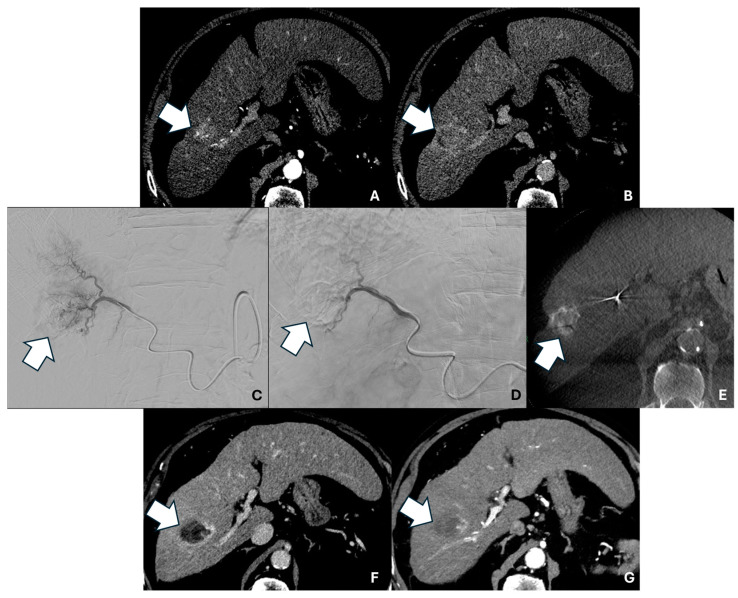
Axial contrast-enhanced pre-procedural CT scan in the arterial (**A**) and delayed (**B**) phases showing a 27 mm HCC nodule in the VII segment (arrows). (**C**) Digital subtraction angiography (DSA) image showing superselective microcatheterization of the HCC lesion with pre-TACE tumor staining. (**D**) Post-TACE superselective DSA image. (**E**) Post-TACE unenhanced cone-beam CT acquisition showing iodinated contrast medium and microsphere uptake by the lesion, with complete coverage (arrow). Axial contrast-enhanced 1 month post-TACE CT scan in the arterial (**F**) and delayed (**G**) phases showing complete response (arrows).

Figure 1 shows the pre-, intra-, and post-procedural imaging of a patient with CR.

After a multidisciplinary tumor board evaluation 1 month post-TACE, of the 11 patients with a 1-month follow-up, 5/11 (45.5%) of them received another treatment: 2 patients (40%) underwent a second TACE session, 1 patient (20%) underwent transarterial radioembolization, 1 patient (20%) had a liver resection, and 1 patient (20%) underwent liver transplantation.

### 2.3. Safety

**Figure 2:**
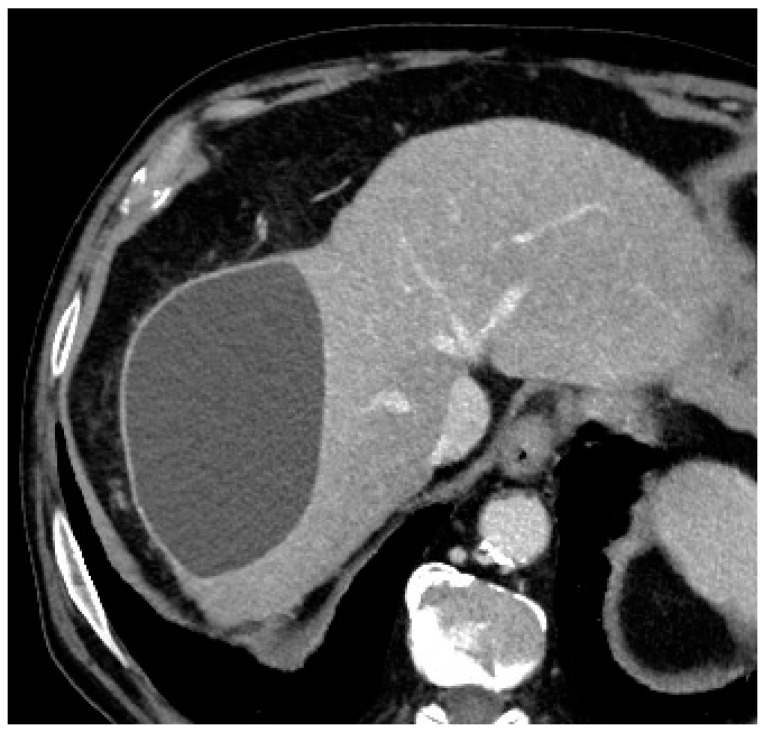
Axial contrast-enhanced CT scan showing an 11 cm × 7 cm × 7 cm biloma in the VII hepatic segment at the 1-month post-procedural follow-up evaluation.

In terms of complications, graded according to the National Cancer Institute Common Terminology Criteria for Adverse Events (NCI-CTCAE) version 3.0, only one (7.7%) intra-procedural complication was registered, consisting of microcatheter blockage during microsphere injection, with no threat to the patient’s health (grade 0 according to NCI-CTCAE) [12]. This blockage was completely and promptly resolved by performing a careful and slow flushing of saline through the microcatheter until a correct flow was restored inside the microcatheter and inside the target vessel. At the 1-month follow-up imaging, two patients (15.4%) had a progressively increasing biloma (Figure 2), which required additional treatment (grade 3 according to NCI-CTCAE): in particular, one patient underwent percutaneous drainage by an interventional radiologist, while the other patient underwent surgical resection of the biloma.

Blood analyses performed at the 1-month post-TACE showed a significant drop in the alpha-fetoprotein (AFP) values, reflecting the therapeutic effect of the chemotherapeutic drug administration during TACE. Regarding the other blood parameters, only a mean significant increase in the alkaline phosphatase (ALP) value was registered at 1 month, with no clinical correlation, while the other blood parameters did not change significantly after TACE. Table 2 reports the mean values of the assessed blood parameters. 

## 3. Discussion

Today, a wide range of treatment options are available for HCC patients [2]. Unfortunately, surgical techniques such as liver resection and liver transplantation may not be possible for a great number of HCC patients. For this reason, locoregional treatments are, as of today, largely adopted and represent an important weapon in fighting HCC. Locoregional treatment options range from curative ones—such as ablative techniques, which sometimes cannot be proposed due to lesion location (i.e., near-critical liver structure or adjacent to other organs), lesion number, or high bleeding risk—to palliative ones; in particular, TACE is an extensively diffuse procedure worldwide, useful in intermediate-stage HCC and also serving as a bridge to surgical resection or liver transplantation in early-stage HCC patients [2]. The technological improvements of the TACE technique have led to the introduction of different types of new DEBs, each made of different materials, and with peculiar characteristics. Currently, there are six commercially available DEBs with CE-marking. Five of these DEBs are non-degradable, permanent, and consist of sulphonate polyvinyl alcohol hydrogel microspheres (DC-Bead), poly(methyl-methacrylate) microspheres (Embozene), acrylic copolymer microspheres (HepaSphere), polyethylene-glycol microspheres (LifePearl®), and microspheres incorporating a radiopaque agent (DC-Bead LUMI) [3,4,5,6,7]. Only one type of DEB is biodegradable and resorbable, the BioPearl^TM^ microspheres: the degradation process of these microspheres is driven by the presence of an aqueous medium that grants a continuous resorption process while releasing a local, constant, sustained, and controlled dose of the chemotherapeutic drug in the target neoplastic lesion. 

Despite the well-established techniques of TACE and the introduction of new bead types, hepatic injury and liver failure are still the most common side effects of TACE treatment. As liver function plays a crucial role in determining the survival of HCC patients undergoing TACE treatment, maintaining an adequate liver function both during and after TACE treatments is essential to ensure that future treatment options, such as surgery or systemic or intra-arterial locoregional therapies, can remain available if needed.

The degradation process that the BioPearl^TM^ microspheres go through allows a progressive restoration of the blood flow to the tumor lesion, with arterial vessels that remain patent for future TACE or other intra-arterial treatments. Several papers have been published on the safety and efficacy of DEB-TACE for HCC treatment, describing the low complication rates as well as the good efficacy profile for patients with HCC in the early and intermediate stages [2,3,4,5,6,7]. However, no data are available on the use of BioPearl^TM^ microspheres.

A prospective, single-arm, multicentric trial, whose study design has been described by Verset and colleagues, is ongoing, with the aim to prospectively assess the feasibility, safety, and technical success of TACE with BioPearl^TM^ microspheres loaded with doxorubicin [13]. Our study retrospectively reports the first results on the safety and feasibility of TACE performed with BioPearl^TM^ microspheres for the treatment of patients with HCC in the early and intermediate stages (BCLC stages A and B).

No intra- nor peri-procedural minor or major complications were observed during the TACE procedure with BioPearl^TM^ microspheres; in particular, no symptoms of postembolization syndrome were reported by the patients. One case (7.7%) of intra-procedural microcatheter blockage was observed, with no consequences on the patient’s health (NCI-CTCAE grade 0), and promptly solved. This blockage could be due to the size of the microspheres (200 μm, range 100–350 μm) as well as to the characteristics of the size and characteristics of the specific target vessel. This could usually be prevented by slowly injecting the microspheres (up to 1 mL per minute) and frequently flushing the system with normal saline after each particle injection. In terms of laboratory tests, the blood analyses performed 1 month after the TACE procedure showed a significant reduction in the alpha-fetoprotein values, consistent with the death of the HCC cells and with a biological response to the chemoembolization treatment. It is worth noting that, regarding the other parameters, only a significant increase in the mean value of alkaline phosphatase at 1 month has been registered, without relevant clinical signs or symptoms; this value could be related to the small size of the population sample.

This study, therefore, shows data on peri- and post-procedural safety that are comparable to those observed in the literature regarding TACE performed with other types of DEBs: as a comparison, the DC-BEADS LUMI microspheres showed no grade 4 and 5 adverse events, whereas the LifePearl® microparticles showed 13.4% of adverse events greater than grade 3, mostly related to postembolization syndrome [2,5,6]. On the other hand, DSM-TACE was reported to have postembolization syndrome rates up to 73.7% [4]. At post-procedural follow-up, we found only two cases (15.4%) of grade 3 complications, consisting, in two cases, of biloma formation, which required additional treatment. When comparing our results to the ones obtained with other microparticles, de Baere and colleagues reported an incidence of bilomas in 9 of 187 DEB-TACE treatments with LifePearl^®^ (4.8%); this difference could be due to the low number of patients in our study [6]. On the other hand, Veloso Gomes and colleagues found 12.6% of patients with hepatic damage after TACE with LifePearl^®^, consisting of bilomas and other procedure-related liver injuries [14]. Moreover, regarding the follow-up of DSM-TACE, it was reported that there was a low rate of incidence of major complications [4].

Concerning the efficacy of BioPearl^TM^ microspheres, the 1-month overall disease control (ODC) and objective response rate (ORR) was 90.9%, with six patients (54.5%) experiencing a CR to HCC treatment after a single TACE procedure. These data are comparable to those reported with the DC-BEAD LUMI microspheres (79.8% of ORR at 1 month and 92.8% of ODC at 1 month) and the LifePearl^®^ microspheres (81% of ORR and 99% of ODC) [5,6]. Orlacchio and colleagues, using the DSM-TACE, experienced a high ORR (84.3% of patients with CR or PR) [4]. The main limitation of this study is the low number of patients enrolled, as well as its retrospective nature, which can hinder the data collection. However, as this is a retrospective and preliminary report on the safety and efficacy of BioPearl^TM^ microspheres, this sample size seems to be enough [15]. Further multicentric studies with a larger sample, a control group, and a longer follow-up are necessary in order to further confirm these preliminary results. Longer follow-ups are also needed in order to confirm the feeding artery patency in the case of TACE retreatments. Hopefully, the aforementioned prospective multicentric trial on BioPearl^TM^ microparticles will shed light on some unanswered questions arising from our preliminary report [13].

## 4. Materials and Methods

This was a retrospective observational study of the feasibility and tolerability of TACE using BioPearl^TM^ microspheres (Terumo Europe NV, Leuven, Belgium) preloaded with doxorubicin for the treatment of unresectable HCC lesions. Thirteen consecutive patients affected by unresectable HCC and treated with DEB-TACE using BioPearls^TM^ microspheres from November 2020 to May 2024 were included in this study. All patients were previously evaluated by a multidisciplinary tumor board composed of specialists involved in the management of HCC (e.g., a hepatologist, hepatobiliary surgeon, transplant surgeon, oncologist, diagnostic and interventional radiologist, and radiation therapist), which gave indication to TACE treatment. Inclusion criteria were the following: age greater than 18 years, histologically confirmed diagnosis of HCC, Eastern Cooperative Oncology Group Performance Status (ECOG-PS) ranging between 0 and 2, tumor size evaluable according to the mRECIST criteria at contrast-enhanced computed tomography (CT) or magnetic resonance imaging (MRI) [11]. Exclusion criteria were the following: previous systemic therapy for HCC, previous intra-arterial treatment, advanced liver disease (Child–Pugh class B or C), advanced neoplastic disease (BCLC stage C or D), history of another primary tumor, portal vein thrombosis, porto-systemic shunt, contraindication to CT and MRI examinations, contraindication to treatment with doxorubicin, or pregnant or breast-feeding women. The study was conducted according to the guidelines of the Declaration of Helsinki and its amendments and was approved by the Institutional Review Board (Comitato Etico Territoriale–CET Lombardia 4) as a retrospective registry (INT 201/23). All the participants signed an informed consent to the procedure.

### 4.1. TACE Protocol

All TACE treatments were performed in a fully equipped angio-suite with all the characteristics of an operatory theater by an interventional radiologist experienced in interventional oncology procedures after antibiotic prophylaxis, during the continuous monitoring of patient vital signs, and during anesthesiological assistance. Pre-operative CT or MRI was evaluated to assess tumor vascularization, identify the feeding artery (or arteries), and exclude arterial anatomical variants. All treatments were performed under local anesthesia (10 mL of 2% mepivacaine), through a right common femoral artery access, with the Seldinger technique, and using a 5 French vascular introducer sheath. Using a 5 French diagnostic catheter and a hydrophilic guidewire, a selective catheterization of the celiac trunk and of the common hepatic artery was performed, and a selective angiography was performed in order to map liver vascularization, identify arteriovenous shunts, assess the feeding arteries of the target HCC lesion, and identify possible branches to non-target structures. Therefore, the distal tract of the segmental hepatic artery feeding the target HCC lesion was then superselectively catheterized using a coaxial technique with a 2.7 French microcatheter (Progreat; Terumo, Tokyo, Japan). Under fluoroscopic guidance, a superselective TACE with the slow infusion of 200 μm BioPearl^TM^ (Terumo Europe NV, Leuven, Belgium) microspheres loaded with doxorubicin (50–100 mg) was performed. BioPearl is a bio-based material, typically derived from biopolymers, such as polylactic acid, kept together by a cross-linker. As the cross-linker degrades, water enters the microspheres and expands them, letting individual components start to degrade. 

The loaded pearls (2 mL of volume) were mixed with 10 mL of non-ionic iodinated contrast solution and 10 mL of distilled water to guarantee the correct volume of infusion. The infusion lasted 10–12 min (median infusion speed of 1 mL/min), checking the beads’ distribution continuously, until the complete administration of the doxorubicin or until obtaining a “stop flow” in the feeding arterial vessel. Cone-beam CT (CBCT) acquisitions, with or without iodinated contrast medium injection, were performed on a case-by-case decision, based on the operator’s preference, to confirm the correct position of the microcatheter in the feeding vessel prior to the administration of the microspheres, to check the correct and full coverage of the HCC lesion after microspheres infusion, and to exclude non-target embolization.

### 4.2. Safety and Efficacy

Safety, evaluated as the occurrence of major and/or minor intra-, peri-, and post-procedural complications, was monitored according to the NCI-CTCAE version 3.0 [12]. Intra-, peri-, and post-procedural morbidity and mortality, consisting of minor and major complications, or a patient’s death, were registered. The mRECIST criteria were used for tumor response assessment at the 1-month follow-up [11]. Contrast-enhanced abdomen and pelvis CT or MRI scans were performed 1 month after the TACE procedure to check the procedural results and assess for late complications. Blood analyses were performed 1 month post-TACE. In case of intra-, peri-, or early post-procedural complications, laboratory tests were performed to assess complete blood count and liver function. Patient survival and tumor control were recorded from the treatment date to the date of tumor progression, of a patient’s death, or to the last available follow-up.

### 4.3. Statistical Analysis

Data of the whole sample (*n* = 13) were analyzed with Stata 18 (StataCorp, College Station, TX, USA). Continuous data were reported as means ± standard deviation (SD). Proportions were expressed in percentage. Chi-square and Student’s *t*-test were used to assess the significance of continuous variables (*p* < 0.05).

## 5. Conclusions

Our preliminary data suggest that transarterial chemoembolization with BioPearl^TM^ microparticles is safe and feasible for the treatment of HCC as indicated by good tolerability.

## Figures and Tables

**Table 1:** Patient characteristics.

	*n*	%
**Age (years; mean ± SD)**	74.6 ± 9.8	
**Gender**		
Female	2	15.4%
Male	11	84.6%
**Child–Pugh class A**	13	100%
A-5	9	69.2%
A-6	4	30.8%
**Etiology of liver cirrhosis**		
Hepatitis B	12	92.3%
Hepatitis C	1	7.7%
**BCLC Stage**		
A	10	76.9%
B	3	23.1%
**Tumor location**		
II	3	14.3%
III	3	14.3%
IV	5	23.8%
V	1	4.8%
VI	4	19%
VIII	5	23.8%
**Maximum lesion diameter**		
<5 cm	11	84.6%
≥5 cm	2	15.4%
**TACE indication**		
Curative	9	69.2%
Down-staging	1	7.7%
Bridging	1	7.7%
Palliation	2	15.4%

SD: standard deviation.

**Table 2:** Blood analyses pre- and post-TACE (1 month).

	Pre-TACE (Mean ± SD)	Post-TACE (Mean ± SD)	*p*
AST (IU/L)	45.0 ± 20.1	43.3 ± 14.1	0.516
ALT (IU/L)	45.7 ± 37.8	37.5 ± 17.7	0.156
ALP (IU/L)	93.8 ± 31.1	140.4 ± 53.5	0.015
GGT (IU/L)	176.3 ± 157.7	168.7 ± 86.9	0.135
Total bilirubin (μmol/L)	1.0 ± 0.3	1.0 ± 0.4	0.905
Direct bilirubin (μmol/L)	0.4 ± 0.2	0.6 ± 0.3	0.369
Albumin (g/L)	4.1 ±0.4	4.0 ± 0.4	0.972
Platelets (×10^3^/μL)	113.1 ± 50.1	138.1 ± 66.8	0.092
White blood cells (×10^3^/μL)	6.3 ± 1.9	7.3 ± 2.3	0.328
Neutrophils (×10^3^/μL)	4.8 ± 1.4	4.9 ± 1.7	0.346
INR	1.1 ± 0.3	1.1 ± 0.1	0.203
Creatinine (mg/dL)	1.0 ± 0.3	1.1 ± 0.5	0.137
Sodium (mEq/L)	137.8 ± 2.6	136.9 ± 2.6	0.212
AFP (ng/mL)	454.9 ± 359.0	24.8 ± 32.8	0.028
MELD 30	8.9 ± 1.1	10.7 ± 3.1	0.282

SD: standard deviation; AST: aspartate-aminotransferase; ALT: alanine-aminotransferase; ALP: alkaline phosphatase; GGT: gamma-glutamyltransferase; INR: International Normalized Ratio; AFP: alpha-fetoprotein; MELD: Model for End-Stage Liver Disease score.

## Data Availability

The raw data supporting the conclusions of this article will be made available by the authors upon request.

## Abbreviations

The following abbreviations are used in this manuscript.

HCC	Hepatocellular Carcinoma
TACE	Transarterial Chemoembolization
cTACE	Conventional TACE
DEB-TACE	Drug-Eluting Beads TACE
ECOG-PS	Eastern Cooperative Oncology Group Performance Status
mRECIST	Modified Response Evaluation Criteria in Solid Tumors
CT	Computed Tomography
MRI	Magnetic Resonance Imaging
CBCT	Cone-Beam CT
NCI-CTCAE	National Cancer Institute—Common Terminology Criteria for Adverse Events
SD	Standard Deviation
BCLC	Barcelona Clinic Liver Cancer
CR	Complete Response
PR	Partial Response
PD	Disease Progression
ODC	Overall Disease Control
ORR	Objective Response Rate
DSA	Digital Subtraction Angiography
AFP	Alpha-fetoprotein
ALP	Alkaline Phosphatase
AST	Aspartate-aminotransferase
ALT	Alanine-aminotransferase
GGT	Gamma-glutamyltransferase
INR	International Normalized Ratio
MELD	Model for End-Stage Liver Disease
